# Five-Year Outcome After Continuous Flow LVAD With Full-Magnetic (HeartMate 3) Versus Hybrid Levitation System (HeartWare): A Propensity-Score Matched Study From an All-Comers Multicentre Registry

**DOI:** 10.3389/ti.2023.11675

**Published:** 2023-09-04

**Authors:** Alessandra Francica, Antonio Loforte, Matteo Attisani, Massimo Maiani, Attilio Iacovoni, Teodora Nisi, Marina Comisso, Amedeo Terzi, Michele De Bonis, Igor Vendramin, Massimo Boffini, Francesco Musumeci, Giovanni Battista Luciani, Mauro Rinaldi, Davide Pacini, Francesco Onorati

**Affiliations:** ^1^ Division of Cardiac Surgery, University Hospital of Verona, Verona, Italy; ^2^ Division of Cardiac Surgery, S. Orsola University Hospital, IRCCS Bologna, Bologna, Italy; ^3^ City of Health and Science Hospital, Cardiac Surgery University Unit, University of Turin, Turin, Italy; ^4^ Division of Cardiac Surgery, Ospedale S. Maria della Misericordia, Udine, Italy; ^5^ Division of Cardiac Surgery, Papa Giovanni XXII Hospital of Bergamo, Bergamo, Italy; ^6^ Division of Cardiac Surgery, IRCCS San Raffaele Hospital, Vita-Salute San Raffaele University, Milan, Italy; ^7^ Division of Cardiac Surgery, San Camillo Forlanini Hospital, Rome, Italy

**Keywords:** continuous-flow LVAD, HeartMate3, HeartWare, full-magnetic levitation pump, hybrid levitation system pump

## Abstract

Despite the withdrawal of the HeartWare Ventricular Assist Device (HVAD), hundreds of patients are still supported with this continuous-flow pump, and the long-term management of these patients is still under debate. This study aims to analyse 5 years survival and freedom from major adverse events in patients supported by HVAD and HeartMate3 (HM3). From 2010 to 2022, the MIRAMACS Italian Registry enrolled all-comer patients receiving a LVAD support at seven Cardiac Surgery Centres. Out of 447 LVAD implantation, 214 (47.9%) received HM3 and 233 (52.1%) received HVAD. Cox-regression analysis adjusted for major confounders showed an increased risk for mortality (HR 1.5 [1.2–1.9]; *p* = 0.031), for both ischemic stroke (HR 2.08 [1.06–4.08]; *p* = 0.033) and haemorrhagic stroke (HR 2.6 [1.3–4.9]; *p* = 0.005), and for pump thrombosis (HR 25.7 [3.5–188.9]; *p* < 0.001) in HVAD patients. The propensity-score matching analysis (130 pairs of HVAD vs. HM3) confirmed a significantly lower 5 years survival (41.7% vs. 64.1%; *p* 0.02), freedom from haemorrhagic stroke (90.5% vs. 70.1%; *p* < 0.001) and from pump thrombosis (98.5% vs. 74.7%; *p* < 0.001) in HVAD cohort. Although similar perioperative outcome, patients implanted with HVAD developed a higher risk for mortality, haemorrhagic stroke and thrombosis during 5 years of follow-up compared to HM3 patients.

## Graphical Abstract

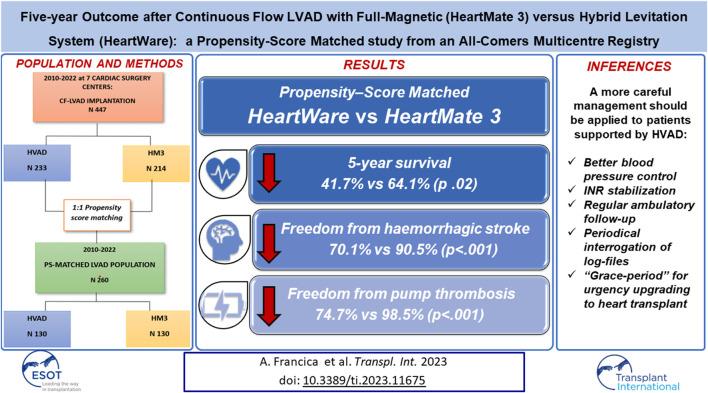

## Introduction

Improved outcomes and increased durability and applicability of long-term mechanical circulatory support have settled this treatment as an effective option for patients with advanced heart failure not suitable for heart transplant. Moreover, donor organ shortage caused a growing interest in Left Ventricle Assist Devices (LVAD) not only as a Bridge-To-Transplant (BTT), but also as destination therapy (DT). In this scenario, continuous-flow pumps have become a standard of care for end-stage heart failure and are currently regarded as the gold standard in LVAD therapy [1]. The HeartWare Ventricular Assist Device (HVAD) by Medtronic and the HeartMate 3 (HM3) by Abbott represents the third-generation centrifugal-flow LVADs (CF-LVADs) implanted worldwide during the last years. The ENDURANCE trial [2] showed the non-inferiority of HVAD versus previous axial-flow pumps, whereas the MOMENTUM-3 trial [3] demonstrated the superiority of the HM3 to the axial-flow Heartmate-II (HMII) in terms of survival and device-related complications. However, on June 2021, HVAD global production and distribution was withdrawn, due to an increased incidence of all-cause mortality and stroke; moreover, several pump failures without an identified cause were reported worldwide [4–6]. Despite its discontinuation, hundreds of patients are still on HVAD. Very limited data exist comparing outcomes with both devices, and previous studies mainly focused on short-term results. Therefore, it is the aim of this study to analyse 5 year survival and freedom from major complications in our Italian all-comer population supported with HVAD or HM3.

## Patients and Methods

### Study Population

From June 2010 to December 2022, the Multicenter Italian Study on Radial Mechanically Assisted Circulatory Support (MIRAMACS) Registry [7] enrolled all-comer adult patients (>18 years of age) requiring LVAD support for end-stage heart failure at seven experienced Cardiac Surgery Centres. Only patients receiving HM3 (Abbott, Chicago, IL, United States) or HVAD (Medtronic, Minneapolis, MN, United States) devices were included in the analysis. All patients with biventricular VADs, isolated right ventricular assist device (RVAD), or axial -flow pumps were excluded. Pre-, intra- and post-operative data were collected. Five-years follow-up was prospectively conducted for all participants, through outpatient visits or direct phone contact to the patient or the referring cardiologist. All data were collected in a dedicated datasheet with predefined variables shared among the Participating Centres. All patient’s data were anonymized with a code of serial numbers. Each Centres had a Principal Investigator and a Collaborator who checked and granted for the anonymization and for the completeness of data. The datasheets from each Centre were then merged in a single database.

The study was conducted according to the guidelines of the Declaration of Helsinki and approved by the “Area Vasta Emilia Centro della Regione Emilia-Romagna” Ethical Committee, “Azienda Ospedaliero—Universitaria di Bologna, Policlinico S. Orsola-Malpighi” (n° 990/2020/Oss/AOUBo; date of approval: 19/11/2020).

### Endpoints

Five-year survival in patients supported by HM3 and HVAD was the primary endpoint of the study. Predictors of survival and 5 years freedom from major adverse events (ischaemic and haemorrhagic stroke, thrombosis, right ventricular failure, gastrointestinal bleeding, and driveline infection) were secondary endpoints. Perioperative outcomes were also assessed. A sub analysis between the first 50 patients implanted with HVAD and the first 50 patients implanted with HM3 was performed in order to investigate a potential learning curve effect. Finally, two sub-analyses were also conducted in patients requiring LVAD as a Destination Therapy (DT) or as a Bridge-To-Transplant (BTT).

Early and late adverse events were defined according to the latest ISHLT definition of adverse events for trials and registries of mechanical circulatory support [8].

### Statistical Analysis

The STROBE checklist was used for reporting observational studies [9]. Descriptive statistics were used to analyse data. Continuous variables are reported as mean ± standard deviation or median (interquartile range), and categorical variables are reported as counts and percentages. Differences between groups were assessed using one-way ANOVA for continuous variables. Categorical data were compared between groups using Pearson’s χ^2^ tests or Fisher’s exact tests, as appropriate. Time-to-event analysis was performed. Kaplan–Meier curves were estimated for mortality and each late adverse event. Differences between groups were assessed by the Log-Rank test. A Cox-regression analysis adjusted for major confounders was used to derive the hazard ratios (HR) and 95% confidence intervals (CI). A multivariate Cox-logistic regression was performed to assess predictors of survival among preoperative and post-operative factors in both HVAD and HM3 population. To account for imbalances between the two cohorts, a propensity score was calculated by logistic regression considering the statistically significant differences among preoperative variables. The Propensity-Score Matching (PSM) was conducted using greedy nearest neighbour matching with a 0.01 caliper and a 1:1 match ratio. The Standardized Mean Differences (SMD) were calculated to assess balance after PSM. The statistical analysis was performed using SPSS Version 27.0 (Armonk, NY, IBM Corp.). A *p*-value of <0.05 was considered statistically significant.

## Results

### Overall Population

**FIGURE 1:**
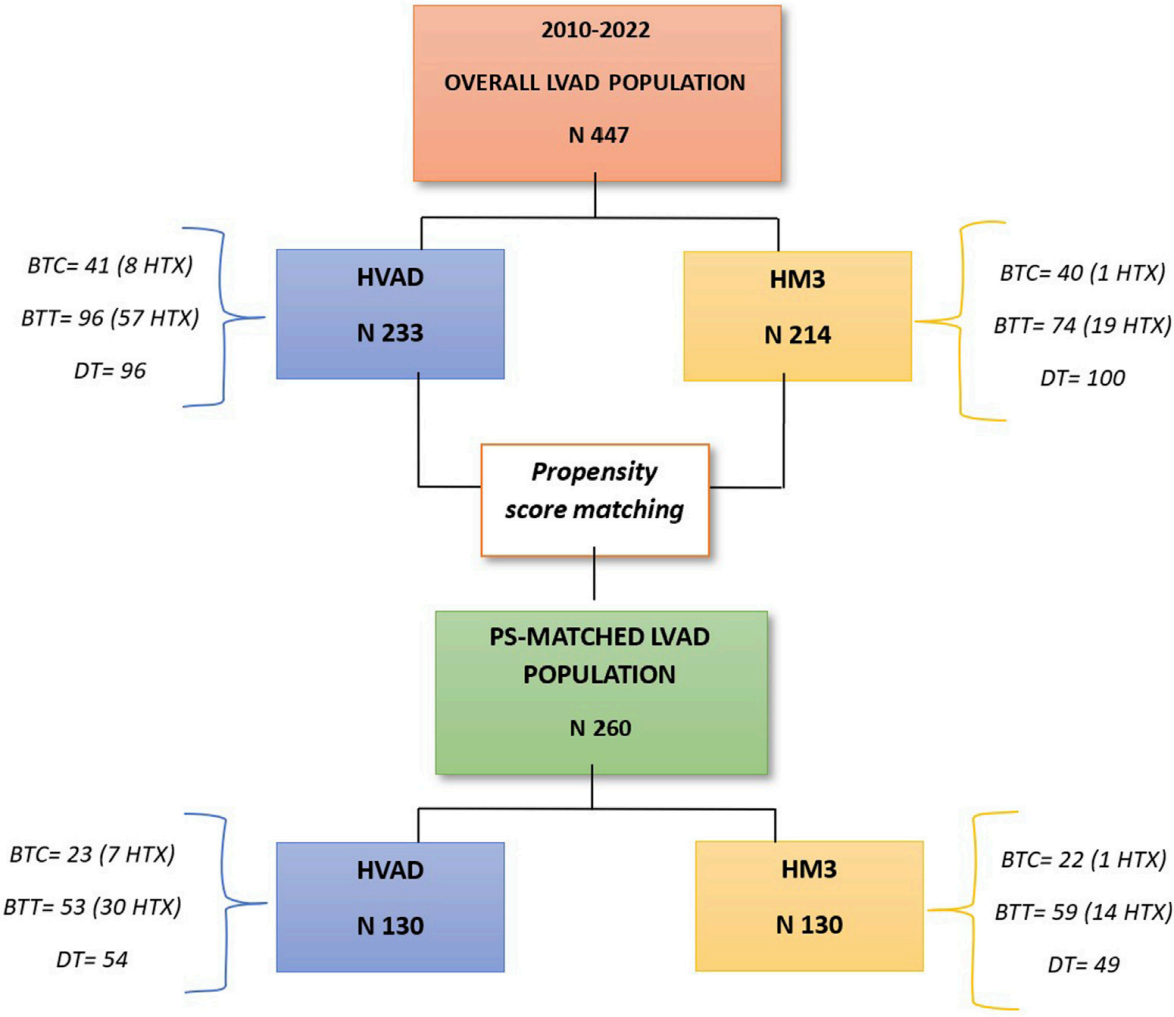
Flowchart of the study population.

Between June 2010 and December 2022, a total of 447 patients were implanted with CF-LVADs at seven Italian Cardiac Surgery Centres: 214 patients (47.9%) received the HM3 and 233 patients (52.1%) received the HVAD (See Figure 1). The two populations differed in several preoperative characteristics. Patients receiving HVAD were younger, with a smaller body surface area and greater preoperative hepatic injury, when compared with HM3 recipients. On the other hand, HM3 patients presented a higher systolic pulmonary arterial pressure, and a more advanced renal impairment than HVAD patients (Supplementary Table S1). Fifty per cent of both populations was in INTERMACS 3, while 10% in INTERMACS 1. Ischemic heart disease and idiopathic cardiomyopathy represented the main indications in both groups, while hypertrophic cardiomyopathy was more observed in HM3 patients (Supplementary Table S1). Periprocedural mortality (14% vs. 9% for HM3 vs. HW; *p* = 0.1) was comparable between the two populations. Detailed hospital outcomes data were reported in Supplementary Table S2.

**FIGURE 2:**
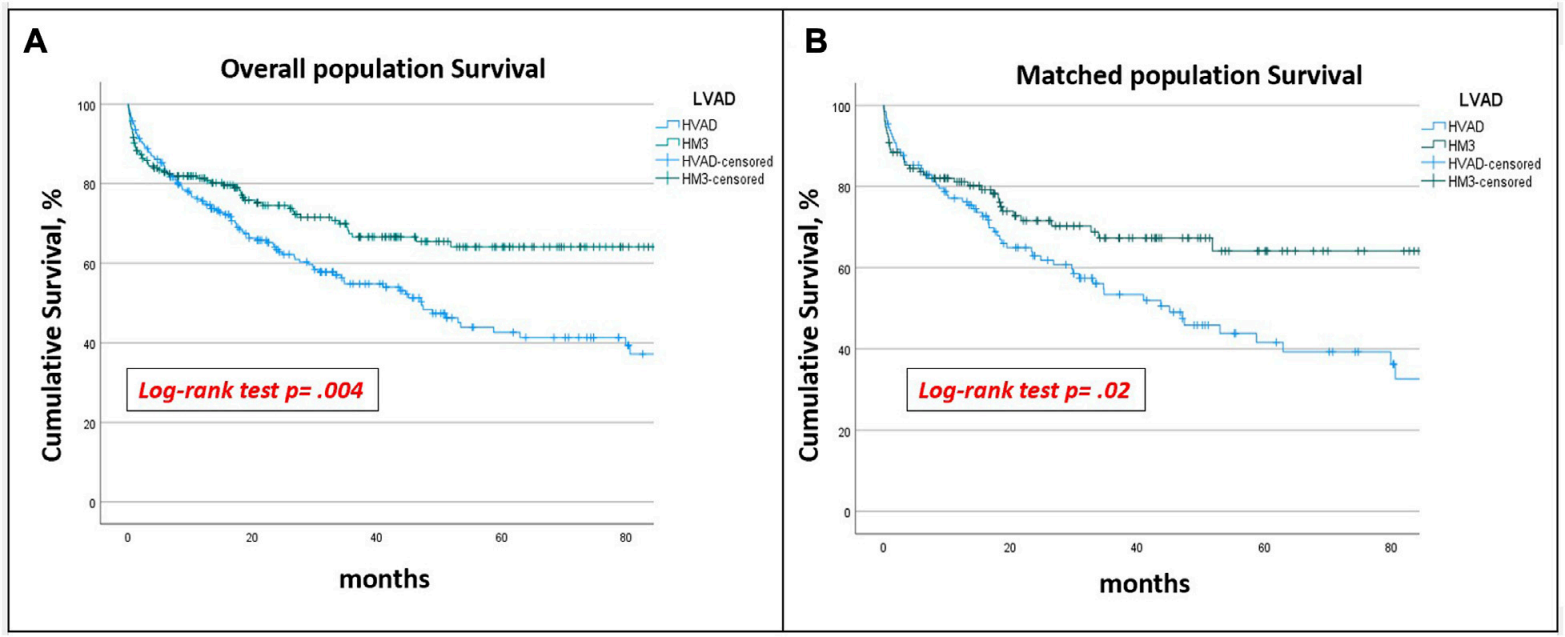
Overall **(A)** and PS-matched survival **(B)**: HVAD patients had a significantly lower 5 years survival than HM3 patients in both unmatched and matched populations.

The mean follow-up time was 65.7 ± 3.1 months. The overall survival at 5 years was higher in HM3 patients (64.1% vs. 42.6%, *p* = 0.004) (Figure 2A). In HVAD cohort, age (HR 1.03 [1.003–1.057]; *p* = 0.028), post-operatively dialysis (HR 2.7 [1.53–4.79]; *p* < 0.001) and ischaemic stroke (HR 2.87 [1.16–7.1]; *p* = 0.023) resulted risk factors for mortality at follow-up (Table 1). In HM3 cohort, preoperative creatinine level (HR 1.46 [1.03–1.2.07]; *p* = 0.032), post-operatively dialysis (HR 1.99 [1.077–3.67]; *p* < 0.03), ischaemic stroke (HR 7.24 [3.4–15.6]; *p* < 0.001) and right ventricular failure (HR 2.96 [1.62–5.43]: *p* < 0.001) resulted risk factors for mortality at follow-up (Table 2).

**TABLE 1:** Independent determinants of survival in HVAD patients.

Independent determinants of survival in HVAD patients			
Preoperative and postoperative factors	HR	95% confidence interval	*p-value*
Age	1.03	1.003–1.06	*0.028*
Post-operative dyalisis	2.7	1.53–4.79	*<0.001*
Post-operative ischaemic stroke	2.87	1.16–7.1	*0.023*

*Statistically significant.

**TABLE 2:** Independent determinants of survival in HM3 patients.

Independent determinants of survival in HM3 patients			
Preoperative and postoperative factors	HR	95% confidence interval	*p-value*
Preoperative creatinin level	1.46	1.03–2.07	*0.032*
Post-operative dyalisis	1.99	1.08–3.67	*0.03*
Post-operative ischaemic stroke	7.24	3.35–15.6	*<0.001*
Post-operative right ventricular failure	2.96	1.62–5.43	*<0.001*

*Statistically significant.

HVAD patients reported a significantly lower freedom from both haemorrhagic (88.6% vs. 69.8%; *p* < 0.001) and ischaemic stroke (91.7% vs. 75.1%; *p* = 0.054), and from pump thrombosis (99.1% vs. 76.8%; *p* < 0.001) (Supplementary Figures S1, S2). No statistical differences in 5 years freedom from right ventricular failure and from driveline infection were reported between groups (Supplementary Figure S2). The Cox-regression analysis adjusted for major confounders showed that HVAD patients had a significantly increased risk for mortality (HR 1.5 [1.2–1.9]; *p* = 0.031), for pump thrombosis (HR 25.7 [3.4–188.9]; *p* < 0.001), and for both haemorrhagic stroke (HR 2.6 [1.3–4.9]; *p* = 0.005) and ischemic stroke (HR 2.08 [1.06–4.08]; *p* = 0.033) (Table 1). Five-year freedom from gastrointestinal bleeding was significantly higher in HM3 patients (90.5% vs. 80.2%; *p* = 0.008) (Supplementary Figure S2), though this difference was lost after adjusting for major confounders at Cox-regression analysis (Table 3).

**TABLE 3:** HVAD vs. HM3: Non-adjust and adjusted Cox-regression analysis for major adverse events at follow-up.

Adverse event	Non-Adjusted Cox-regression		Adjusted^a^ Cox-regression	
	HVAD vs. HM3 HR (95% CI)	*p*	HVAD vs. HM3 HR (95% CI)	*p*
Mortality	1.6 [1.16–2.17]	0.004	1.5 [1.2–1.9]	*0.031*
Haemorrhagic stroke	3.04 [1.6–5.8]	<0.001	2.6 [1.3–4.9]	*0.005*
Ischaemic stroke	1.8 [0.97–3.5]	0.058	2.08 [1.06–4.08]	*0.033*
Pump Thrombosis	27.7 [3.8–203.8]	0.001	25.7 [3.4–188.9]	*<0.001*
GI bleeding	2.4 [1.2–4.5]	0.01	1.6 [0.81–3.2]	*0.17*
RV failure	1.14 [0.77–1.7]	0.51	0.96 [0.62–1.4]	*0.83*
DL infection	1.3 [0.88–1.89]	0.2	1.3 [0.85–2.04]	*0.21*

^a^ Adjusted for age, BSA, ALT, creatinine, primary heart disease, sPAP.

DL, driveline; GI, gastrointestinal; RV, right ventricle.

*Statistically significant.

### Heart Transplant, LVAD Explant or Exchange

A total of 65 HVAD (57 BTT and 8 BTC) and 20 HM3 (19 BTT and 1 BTC) patients underwent to heart transplant. Among HVAD patients, 22 (33.8%) underwent to heart transplant because of LVAD complications (14 because of pump thrombosis, 4 because of LVAD infection), eight of whom in urgency tier. Only four patients (one in urgency) in HM3 cohort were transplanted because of LVAD infection. Only two patients underwent to HVAD explant for recovery, while one patient underwent HVAD exchange for pump thrombosis, but died postoperatively. All other patients who experienced thrombosis were pharmacologically treated and 14 of them transplanted.

### Sub-Analysis of the First 50 Cases of HVAD and HM3 Implantation

The sub analysis on the first 50 cases of implantation of HVAD and HM3 confirmed a worse outcome in HVAD patients. Perioperative mortality was higher in HVAD patients compared to HM3, though not statistically significant (10% vs. 4%; *p* = 0.43). Long-term outcome analysis confirmed worse 5 years survival (24.8% vs. 68.1%; *p* < 0.001), lower freedom from haemorrhagic (54.5% vs. 80.8%; *p* = 0.04) and ischaemic stroke (71.2% vs. 95.8; *p* = 0.007) and from pump thrombosis (62.6% vs. 100%; *p* < 0.001). Five-year freedom from gastrointestinal bleeding (79.4% vs. 90.2%; *p* = 0.12), from right ventricular failure (66.4% vs. 77.9%; *p* = 0.29) and from drive-line infection (54.6% vs. 63.4%; *p* = 0.83) were similar between the two cohorts.

### Propensity Matched Population

**FIGURE 3:**
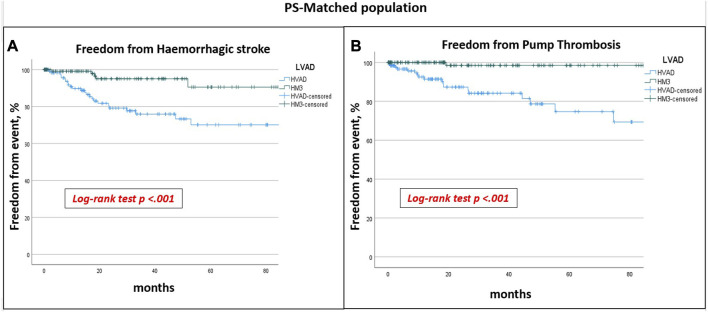
PS-matched population freedom from haemorrhagic stroke **(A)** and from pump thrombosis **(B)**: HVAD patients had a significantly lower freedom from haemorrhagic stroke and from pump thrombosis.

**FIGURE 4:**
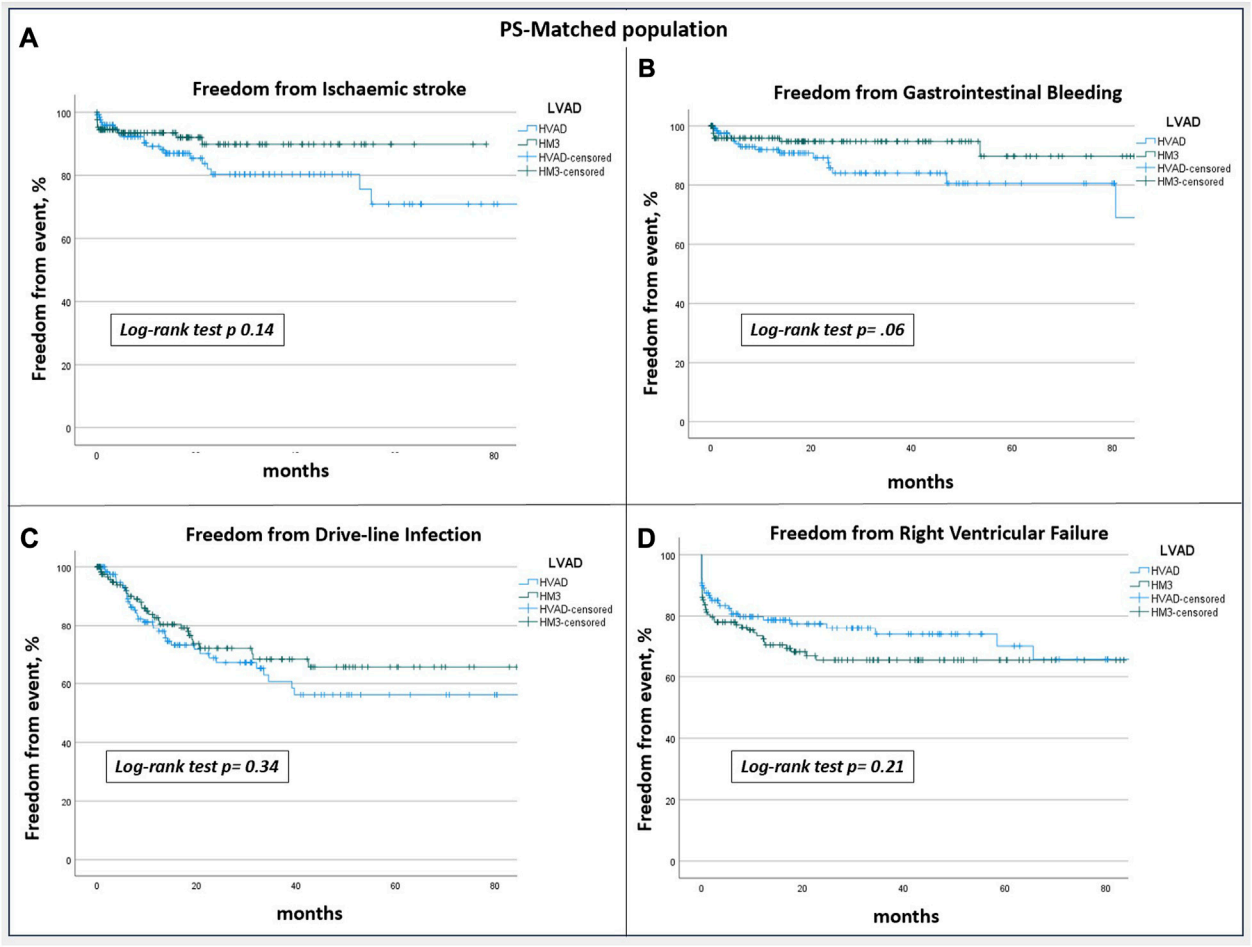
PS-matched populations freedom from **(A)** ischaemic stroke, **(B)** gastrointestinal bleeding, **(C)** driveline infection, **(D)** right ventricular failure: no statistically significant differences at 5 years were found between HVAD and HM3 cohorts.

After PSM-analysis, 130 pairs of patients with similar preoperative profiles receiving HM or HVAD were selected. Preoperative characteristics are reported in Table 4. Post-operative complications remained similar between the groups, with the exception for prolonged ventilation and sepsis which were more frequent in HM3 patients (Table 5). HVAD patients confirmed a significantly lower 5 years survival (64.1% vs. 41.7%; *p* 0.02) (Figure 2B), freedom from haemorrhagic stroke (90.5% vs. 70.1%; *p* < 0.001) and from pump thrombosis (98.5% vs. 74.7%; *p* < 0.001) (Figure 3). Freedom from ischaemic stroke remained lower in HVAD compared to HM3, but non-statistically significant (Figure 4). Freedom from gastrointestinal bleeding, driveline infection and right heart failure were comparable between HM3 and HVAD (Figure 4).

**TABLE 4:** PS-matched population: Preoperative characteristics.

Preoperative characteristics *n* (%), m (SD)	HM3 (n 130)	HVAD (n 130)	*p*	SMD
Age, years	60.2 (8.7)	59.8 (10.5)	*0.71*	*0.04*
Sex, males	118 (90.8)	116 (89.2)	*0.68*	*0.03*
BSA, cm/m^2^	1.9 (0.19)	1.9 (0.17)	*0.56*	*0*
Creatinine, mg/dL	1.4 (0.63)	1.4 (0.49)	*0.86*	*0*
AST, U/L	37.6 (37.9)	32.4 (31.3)	*0.24*	*0.1*
ALT, U/L	34.9 (35.3)	32.6 (26.3)	*0.55*	*0.07*
Atrial fibrillation	52 (24.3)	35 (15)	0.013	
EF, %	20.4 (5.9)	21.1 (7.1)	*0.41*	*0.1*
LVEDV, mL	263.4 (78.5)	261.3 (111.1)	0.9	0.002
TAPSE, mm	16.8 (4.3)	16.7 (4.5)	0.88	0.002
PVR (Fick), wood	3.3 (1.9)	3.5 (2.07)	*0.23*	*0.07*
Cardiac index (Fick)	1.9 (0.54)	1.9 (0.55)	*0.38*	*0*
Heart disease			0.73	*0.07*
Idiopathic	54 (41.5)	60 (46.2)		
Hypertrophic	3 (2.3)	5 (3.8)		
Ischemic	67 (51.5)	60 (46.2)		
Other	6 (2.3)	5 (1.9)		
Intermacs			*0.23*	*0.1*
1	21 (9.2)	7 (5.4)		
2	21 (16.1)	33 (25.4)		
3	68 (52.3)	65 (50)		
4	29 (22.3)	25 (19.2)		
IABP	44 (33.8)	33 (25.8)	*0.14*	*0.1*
VA-ECMO	7 (5.4)	8 (6.2)	*0.8*	*0.03*
REDO	8 (6.2)	10 (7.7)	*0.9*	*0.05*
Indication			*0.74*	*0*.*06*
BTT	59 (45.4)	53 (40.8)		
DT	49 (37.6)	54 (41.5)		
BTC	22 (16.9)	23 (17.7)		

BSA, body surface area; EF, ejection fraction; IABP, intra-aortic balloon pump; INTERMACS, interagency registry for mechanically assisted circulatory support; LVEDV; left ventricular end diastolic volume; PAP, systolic pulmonary arterial pressure; PVR, pulmonary vascular resistance; VA-ECMO, veno-arterial extracorporeal membrane oxygenation.

*Statistically significant.

**TABLE 5:** PS-matched population: In-hospital outcomes.

In-hospital outcome n (%), m (SD)	HM3 (n 130)	HVAD (n 130)	*p*
In-hospital mortality	15 (11.5)	11 (8.5)	*0.41*
CPB time, min	106.3 (37.9)	98.4 (44.4)	*0.16*
Total Implantation time, min	317.72 (85.19)	329 (262.5)	*0.7*
Bleeding requiring surgical revision	16 (12.3)	18 (13.8)	*0.71*
Prolonged ventilation (>72 h)	37 (28.5)	11 (8.5)	*<0.001*
Dialysis	22 (16.9)	13 (10)	*0.1*
Sepsis	46 (35.4)	21 (16.2)	*<0.001*
Ischaemic stroke	7 (5.4)	5 (3.8)	*0.55*
Haemorrhagic stroke	0	0	
Right ventricular failure	27 (20.8)	15 (11.5)	*0.043*
Temporary RVAD	6 (4.6)	4 (3)	*0.8*
ICU days	17.8 (22.1)	15.2 (22.9)	*0.37*
In-hospital days	44.14 (50.1)	39.4 (46.7)	*0.45*

CPB, cardiopulmonary bypass; ICU, intensive care unit; RVAD, right ventricular assist device.

*Statistically significant.

Out of 103 DT patients, 49 received HM3 and 54 received HVAD. More than 80% were male in both groups, with a mean age of 66.2 ± 5.6 in HM3 vs. 67.5 ± 5.02 in HVAD (*p* = 0.18) (Table 6). Post-operative mortality was comparable (8.2% vs. 5.6% in HM3 and HVAD respectively; *p* = 0.7), as well as all post-operative complications, except for right ventricular failure that was more common in HM3 patients (Table 7). The HVAD cohort had lower 5 years cumulative survival (59.9% vs. 37% *p* = 0.03) (Supplementary Figure S3A) and freedom from haemorrhagic stroke (76.7% vs. 65.4%; *p* = 0.01) (Supplementary Figure S3B). In this sub-population, freedom from thrombosis resulted lower in HVAD, though not statistically significant (Supplementary Figure S4). No statistical differences were reported for the other adverse events (Supplementary Figure S5).

**TABLE 6:** PS-matched DT population: Preoperative characteristics.

Preoperative characteristics n (%), m (SD)	HM3 (n 49)	HVAD (n 54)	*p*
Age, years	66.2 (5.6)	67.5 (5.02)	*0.18*
Sex, males	43 (87.8)	46 (85.2)	*0.7*
BSA, cm/m^2^	1.9 (0.16)	1.8 (0.15)	*0.21*
Creatinine, mg/dL	1.6 (0.76)	1.5 (0.45)	*0.65*
AST, U/L	33.09 (34.4)	41.6 (59.4)	*0.39*
ALT, U/L	26.1 (14.9)	38.8 (37.9)	*0.03*
Atrial fibrillation	15(30.6)	12 (22.2)	*0.33*
EF, %	18.9 (6.4)	18.9 (5.7)	*0.98*
LVEDV, mL	247.12 (94.2)	272. 5 (94.2)	0.26
TAPSE, mm	16.9 (4.03)	17.5 (5.3)	0.55
PVR (Fick), wood	2.7 (1.3)	3.1 (2.1)	*0.19*
Cardiac index (Fick)	2.2 (0.65)	2.13 (0.57)	*0.5*
sPAP, mmHg	43.04 (15.5)	40.4 (15.2)	*0.38*
Heart disease			0.41
Idiopathic	16 (32.7)	25 (46.3)	
Hypertrophic	0	0	
Ischaemic	31 (63.3)	28 (51.9)	
Other	1 (2)	1 (1.9)	
Intermacs			*0.21*
1	3 (6.1)	1 (1.9)	
2	7 (14.3)	16 (29.6)	
3	29 (59.9)	29 (53.7)	
4	10 (20.4)	8 (7.8)	
IABP	20 (40.8)	22 (40.7)	*0.99*
VA-ECMO	2 (4.1)	5 (9.3)	*0.44*
REDO	5 (10.2)	4 (7.4)	*0.73*

BSA, body surface area; DT, destination therapy; EF, ejection fraction; IABP, intra-aortic balloon pump; LVEDV; center ventricular end diastolic volume; PAP, systolic pulmonary arterial pressure; PVR, pulmonary vascular resistance; VA-ECMO, veno-arterial extracorporeal membrane oxygenation

*Statistically significant.

**TABLE 7:** PS-matched DT population: In-hospital outcomes.

In-hospital outcome n (%), m (SD)	HM3 (n 49)	HVAD (n 54)	*p*
In-hospital mortality	4 (8.2)	3 (5.6)	*0.7*
CPB time, min	109.7 (35.)	107.03 (49.9)	0.08
Total Implantation time, min	318.6 (87.7)	392 (348.12)	*0.22*
Bleeding requiring surgical revision	4 (7.4)	4 (7.4)	*1*
Prolonged ventilation (>72 h)	13 (26.5)	6 (11.1)	*0.044*
Dialysis	8 (16.3)	4 (7.4)	*0.22*
Sepsis	15 (30.6)	7 (13)	*0.029*
Ischaemic stroke	2 (4.1)	1 (1.9)	*0.6*
Haemorrhagic stroke	0	0	*—*
Right ventricular failure	11 (22.4.6)	3 (5.5)	*0.019*
Temporary RVAD	3 (6.1)	2 (3.7)	*0.8*
ICU days	18.9 (24.4)	17.6 (21.3)	*0.79*
In-hospital days	38.3 (25.3)	33.3 (25.5)	*0.38*

DT, Destination therapy; CPB, cardiopulmonary bypass; ICU, intensive care unit; RVAD, right ventricular assist device.

*Statistically significant.

Out of 116 BTT patients, 59 were supported by HM3 and 53 by HVAD. Time to transplant was shorter in HVAD (36.7 vs. 49.9 months; *p* = 0.019) (Supplementary Figure S6B). The cumulative 5 years survival was comparable between the two cohorts (Supplementary Figure S6A), as well as the freedom from adverse events (Supplementary Figures S7, S8), except freedom from pump thrombosis which was lower in HVAD patients (Supplementary Figure S7B). Preoperative and post-operative data of BTT are displayed in Supplementary Tables S3, S4.

## Discussion

In this Italian multicentre observational study, we compared 5 years survival and freedom from major adverse events in patients supported either by HVAD or HM3. HVAD recipients showed a significantly lower 5 years survival with a higher risk of haemorrhagic stroke and pump thrombosis compared to the HM3 patients, before and after the PSM analysis. Freedom from ischaemic stroke, gastrointestinal bleeding, right heart failure, and driveline infections did not significantly differ between the two groups after PSM. To the best of our knowledge, scanty data exist comparing 5 years outcome of these two different CF-LVADs outside of the industry-driven trials. Furthermore, both devices have been preferentially compared to historical cohorts implanted with the second generation axial-flow pumps [2, 3]. More in detail, few retrospective single-centre studies and three registry-based studies compared HM3 and HVAD, and all reported a higher incidence of adverse events in HVAD patients [10–16]. In line with our results, Mueller et al. [10] and Numan et al. [12] reported a significantly higher incidence of haemorrhagic stroke and pump thrombosis in HVAD patients at 12 and 36 months, respectively, whereas Mihalj et al. [13] reported an increased risk of device malfunctions, though excluding pump thrombosis. However, none of these single-centre studies showed a significant difference in follow-up survival between HM3 and HVAD, but the median follow-up time never exceeded 3 years. Similarly, the EUROMACS analysis by Potapov et al. [14] reported a higher incidence of pump thrombosis and haemorrhagic stroke in HVAD recipients already at 2 years of follow-up, although survival was comparable. However, despite the reported survival of HVAD and HM3 of all the above-mentioned studies was always comparable, the slopes of the curves always addressed a higher survival in the HM3 cohorts, thus highlighting the potential for biases related to the small sample sizes and the short-term follow-up times of these analyses [10–16]. On the contrary, our data agree with the latest report from the STS Intermacs database published by Pagani et al. [16], which identified an important survival benefit at 2 years of follow-up after HM3 implantation compared to HVAD support. Analogous results were also observed by a recent large-scale multicentre study by Numan et al. [17], which confirmed a significantly better survival and a lower occurrence of pump thrombosis for HM3 patients at 2 years of follow-up, in both un-adjusted and adjusted populations. All these results are in line with our findings and suggests that patients on HVAD support have a worse life-expectation than patients on HM3 support, an we also demonstrated that it did not depends by the learning curve time. Indeed, one large multicentre study reported the longest follow-up of HVAD-patients: this study was the only one able to achieve a 6 years freedom from any stroke of 82%, and a freedom from severely disabling stroke of 89% [18], possibly suggesting a better risk-profile and a better patient selection than our and all the above-mentioned studies.

Different from our findings, Numan et al. [17] found no differences in the occurrence of haemorrhagic stroke between HVAD and HM3. Conversely, an in-depth analysis of cerebrovascular adverse events from the INTERMACS registry [15] showed a higher occurrence of both ischaemic and haemorrhagic cerebrovascular adverse events in patients on HVAD support. Similarly, our study reported higher ischaemic and haemorrhagic strokes in the overall population of HVAD patients, although the incidence of ischaemic stroke loses statistical significance after the PSM analysis. The latter finding could be explained by the reduced number of events in the matched cohorts. On the other hand, we observed no differences for gastrointestinal bleeding, driveline infections, and right heart failure, as reported in previous studies [10–16].

When DT subgroup was considered, a higher survival rate and lower incidence of haemorrhagic stroke were still observed in the HM3 cohort when compared with HVAD, in line with a recent single-centre study by Wasilewski et al. [19] who reported a better survival and freedom from complications in HM3 compared to HVAD in DT patients at 2 years of follow-up. Finally, our sub analysis on BTT patients showed that HVAD recipients underwent heart transplant more commonly than HM3. This is explained by the different follow-up time between the two cohorts given that HM3 was launched in the market later than HVAD, as also highlighted in previous studies [14,17], and by the fact that patients on HVAD were transplanted more quickly because of the higher rate of pump thrombosis, thus qualifying for a high urgency tier. Finally, the occurrence of pump thrombosis confirmed to be higher in patients HVAD population. Preemptive replacement of the HVAD by HM3 has shown to reduce survival compared with continued HVAD support [20], resulting in the current recommendation to strict follow-up these patients and to optimize their clinical management. Blood pressure control, INR stabilization with an increased INR point-of-care testing, more regular ambulatory follow-up with periodical interrogation of log-files, echo-guided rump tests, have been all demonstrated to improve survival, reduce stroke, and early detect subclinical thrombosis [21–27]. A recent ISHLT consensus [28] on the management of patients still supported by HVAD better summarized all these key-points, highlighting how a successful long-term management of HVAD patients depends on comprehensive care by a multidisciplinary team. Based on our findings, reporting lower survival, higher stroke, and higher pump thrombosis in HVAD patients, as early as after the first year of follow up, we stigmatize the importance of all the above-mentioned recommendations for the care of these patients. Furthermore, a recently approved new Italian allocation system for heart transplants allows a yearly 1 month “grace-period” (i.e., upgrade to urgency status LVAD-patients with at least 18 months of follow-up who do not reach the standard criteria for urgency/emergency). We therefore suggest that patients on HVAD fulfilling “grace period criteria,” especially if at low- or intermediate-risk for heart transplant, should be deeply considered for the transplant.

### Limitations

The main limitation of the study stems for its non-randomized nature. However, the strength of the study is that confirms over 5 years of follow up findings already reported over shorter time frames. MIRAMACS is the first Italian nation-level observational multicentre registry, gathering all-comer adult patients undergoing third generation CF-LVAD. Therefore, it reports “real-world” data from a wide interinstitutional experience. Though it confirms the worse-life expectation of HVAD patients, it also highlights the good 5 years outcome of HM3 device outside from MOMENTUM-3 data [3].

Another limitation relates to the difference in mean follow-up time between HVAD and HM3, though this unavoidable bias stems from the different marketing time of the two devices. However, Cox regression analysis and PSM analysis were performed to account for possible confounders.

Finally, this study reports a national trend in LVAD policy and management, and unaddressed bias might limit its reproducibility in countries with other allocation systems and policies.

## Data Availability

The dataset will be available on request. Requests to access the datasets should be directed to the last corresponding author.

## Supplementary Material

Supplementary Figure S1 Overall population freedom from haemorrhagic stroke **(A)** and from pump thrombosis **(B)**: HVAD patients had a significantly lower freedom from haemorrhagic stroke and from pump thrombosis.

Supplementary Figure S2 Overall populations freedom from **(A)** ischaemic stroke, **(B)** gastrointestinal bleeding, **(C)** driveline infection, **(D)** right ventricular failure: HVAD showed lower freedom from ischaemic stroke and from gastrointestinal bleeding compared to HM3 patients. No differences were found in freedom from driveline infection and right ventricular failure.

Supplementary Figure S3 PS-matched population: DT 5 years survival **(A)** and freedom from haemorrhagic stroke **(B)**: HVAD patients had a significantly lower survival and freedom from haemorrhagic stroke at 5 years compared to HM3 patients.

Supplementary Figure S4 PS-matched population: DT 5 years and freedom from pump thrombosis **(B)**: no statistically significant differences were found in freedom from pump thrombosis between HM3 and HVAD cohorts.

Supplementary Figure S5 PS-matched populations: DT freedom from **(A)** ischaemic stroke, **(B)** gastrointestinal bleeding, **(C)** driveline infection, **(D)** right ventricular failure: no statistically significant differences at 5 years were found between HVAD and HM3 cohorts.

Supplementary Figure S6 PS-matched population: BTT 5 years survival **(A)** and time to heart transplant **(B)**: HM3 and HVAD patients had comparable survival, but time to transplant was lower for HVAD patients.

Supplementary Figure S7 PS-matched population: BTT freedom from haemorrhagic stroke **(A)** and from pump thrombosis **(B)**: HVAD patients had a significantly lower freedom from pump thrombosis, but not from haemorrhagic stroke.

Supplementary Figure S8 PS-matched populations: BTT freedom from **(A)** ischaemic stroke, **(B)** gastrointestinal bleeding, **(C)** driveline infection, **(D)** right ventricular failure: no statistically significant differences at 5 years were found between HVAD and HM3 cohorts.
