# Corrigendum: Five-Year Outcome After Continuous Flow LVAD With Full-Magnetic (HeartMate 3) Versus Hybrid Levitation System (HeartWare): A Propensity-Score Matched Study From an All-Comers Multicentre Registry

**DOI:** 10.3389/ti.2023.12088

**Published:** 2023-10-09

**Authors:** Alessandra Francica, Antonio Loforte, Matteo Attisani, Massimo Maiani, Attilio Iacovoni, Teodora Nisi, Marina Comisso, Amedeo Terzi, Michele De Bonis, Igor Vendramin, Massimo Boffini, Francesco Musumeci, Giovanni Battista Luciani, Mauro Rinaldi, Davide Pacini, Francesco Onorati

**Affiliations:** ^1^ Division of Cardiac Surgery, University Hospital of Verona, Verona, Italy; ^2^ Division of Cardiac Surgery, S. Orsola University Hospital, IRCCS Bologna, Bologna, Italy; ^3^ City of Health and Science Hospital, Cardiac Surgery University Unit, University of Turin, Turin, Italy; ^4^ Division of Cardiac Surgery, Ospedale S. Maria della Misericordia, Udine, Italy; ^5^ Division of Cardiac Surgery, Papa Giovanni XXII Hospital of Bergamo, Bergamo, Italy; ^6^ Division of Cardiac Surgery, IRCCS San Raffaele Hospital, Vita-Salute San Raffaele University, Milan, Italy; ^7^ Division of Cardiac Surgery, San Camillo Forlanini Hospital, Rome, Italy

**Keywords:** continuous-flow LVAD, HeartMate3, HeartWare, full-magnetic levitation pump, hybrid levitation system pump

In the original article, there was a mistake in the **Graphical Abstract** as published. The propensity-score matching survival was incorrectly given as “64.1% vs. 81.3% (p .02)”. The correct values are “64.1% for HM3 vs. 41.7% in HVAD (p 0.02)”. The Kaplan-Meier curve is correct, it was just a transcription error.

**GRAPHICAL ABSTRACT:**
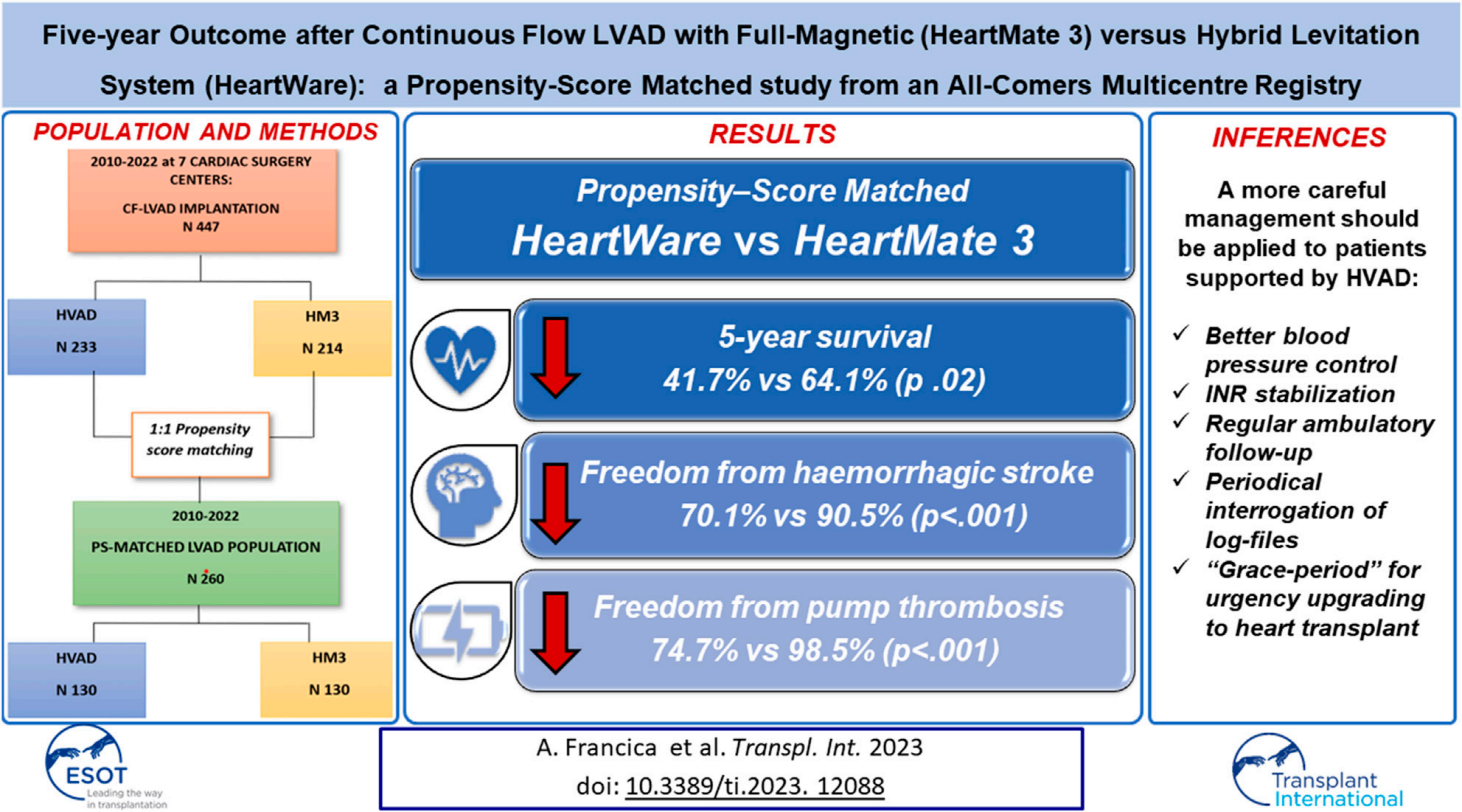


The corrected **Graphical Abstract** appears below.

The same mistake was present in the text body. A correction has been made to the **Abstract** and to the **Results** section:

**Abstract**: “The propensity-score matching analysis (130 pairs of HVAD vs. HM3) confirmed a significantly lower 5 years survival (41.7% vs. 64.1%; *p* 0.02)…”

**Results**, *Propensity Matched Population*: “HVAD patients confirmed a significantly lower 5 years survival (64.1% vs. 41.7%; *p* 0.02) (Figure 2B)…”

The authors apologize for these errors and state that this does not change the scientific conclusions of the article in any way. The original article has been updated.

