# Supplementary figures and images for: Five-Year Outcome After Continuous Flow LVAD With Full-Magnetic (HeartMate 3) Versus Hybrid Levitation System (HeartWare): A Propensity-Score Matched Study From an All-Comers Multicentre Registry
Source: Transpl Int. 2023 Sep 4;36:11675. doi: 10.3389/ti.2023.11675 (PMC10505657; doi:10.3389/ti.2023.11675)

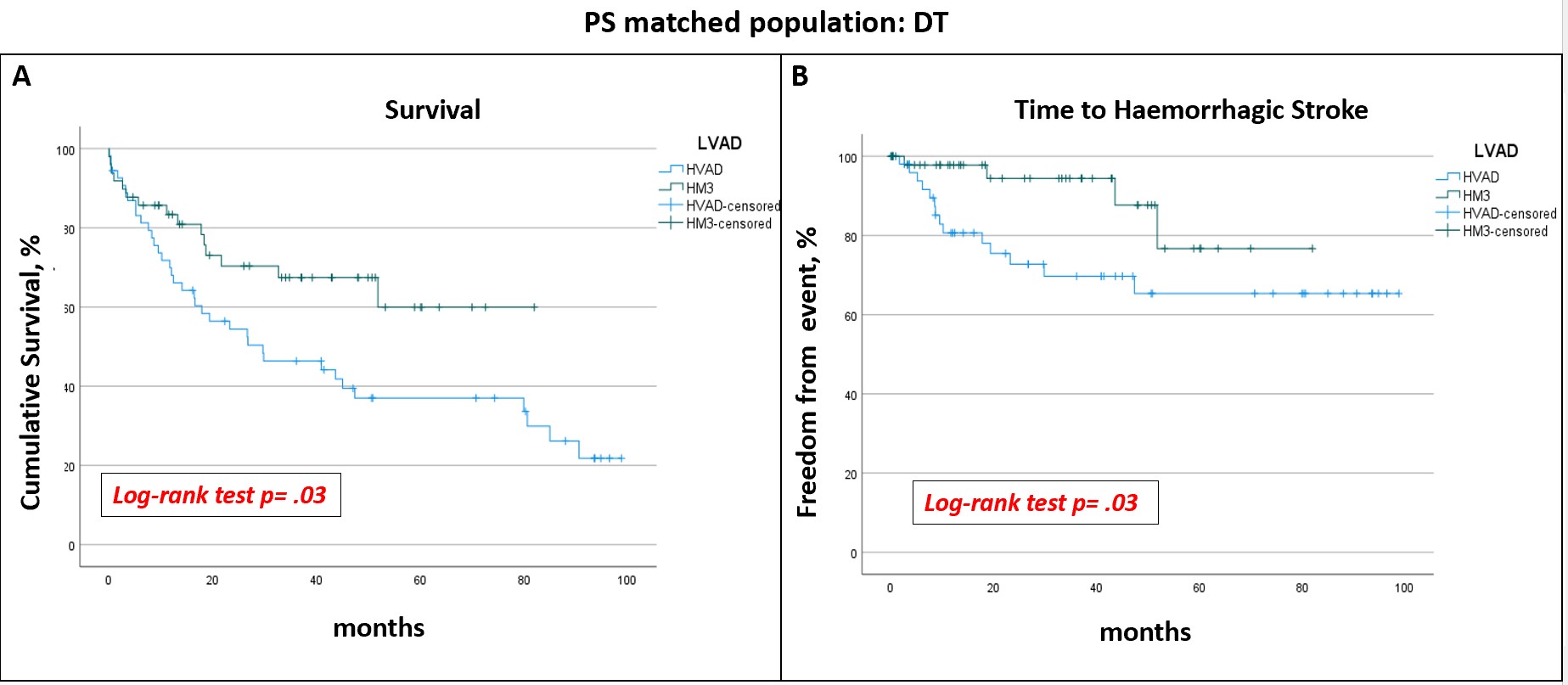

Supplement: Supplementary file 1 [file Image3.JPEG]

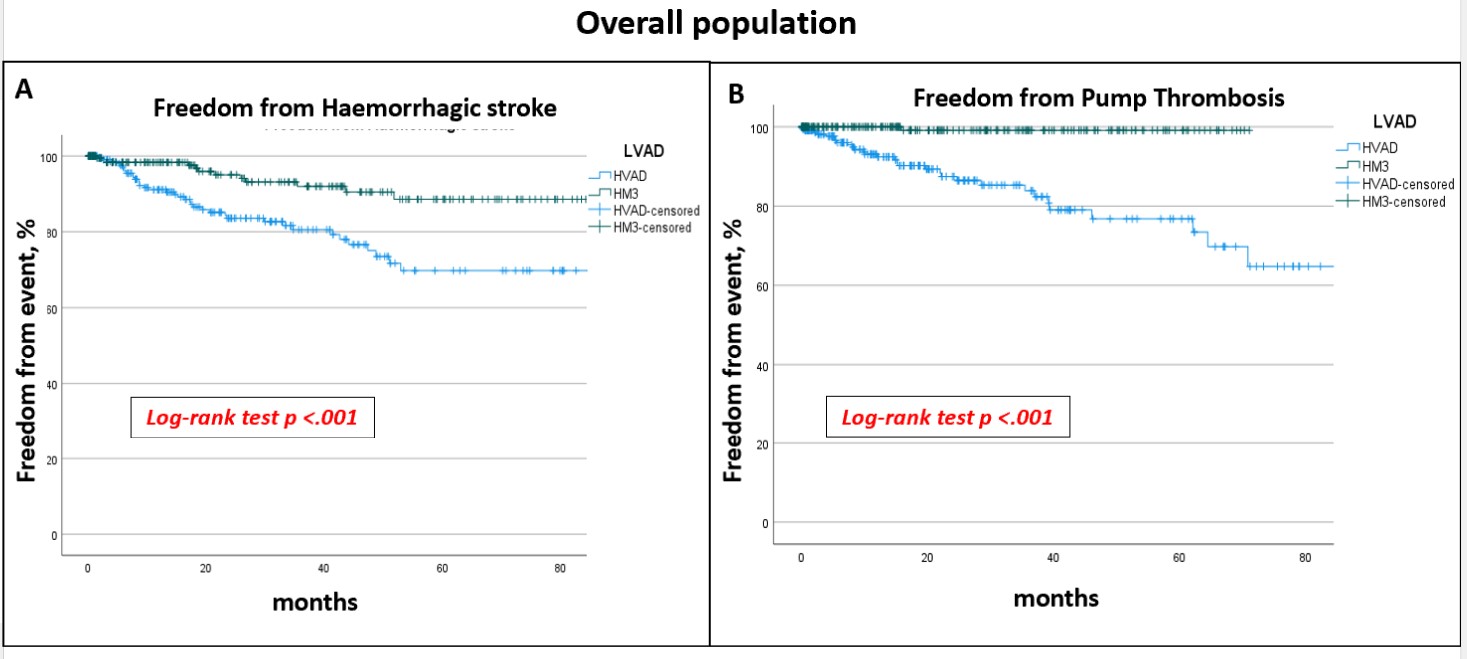

Supplement: Supplementary file 2 [file Image1.JPEG]

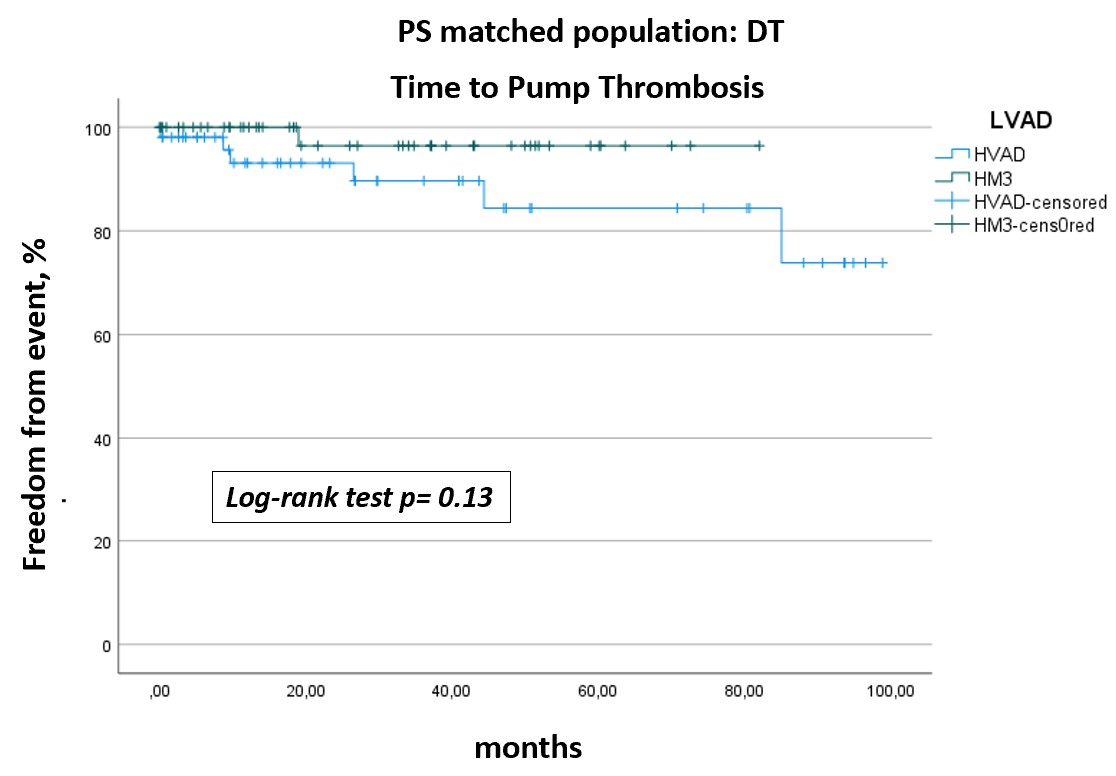

Supplement: Supplementary file 3 [file Image4.JPEG]

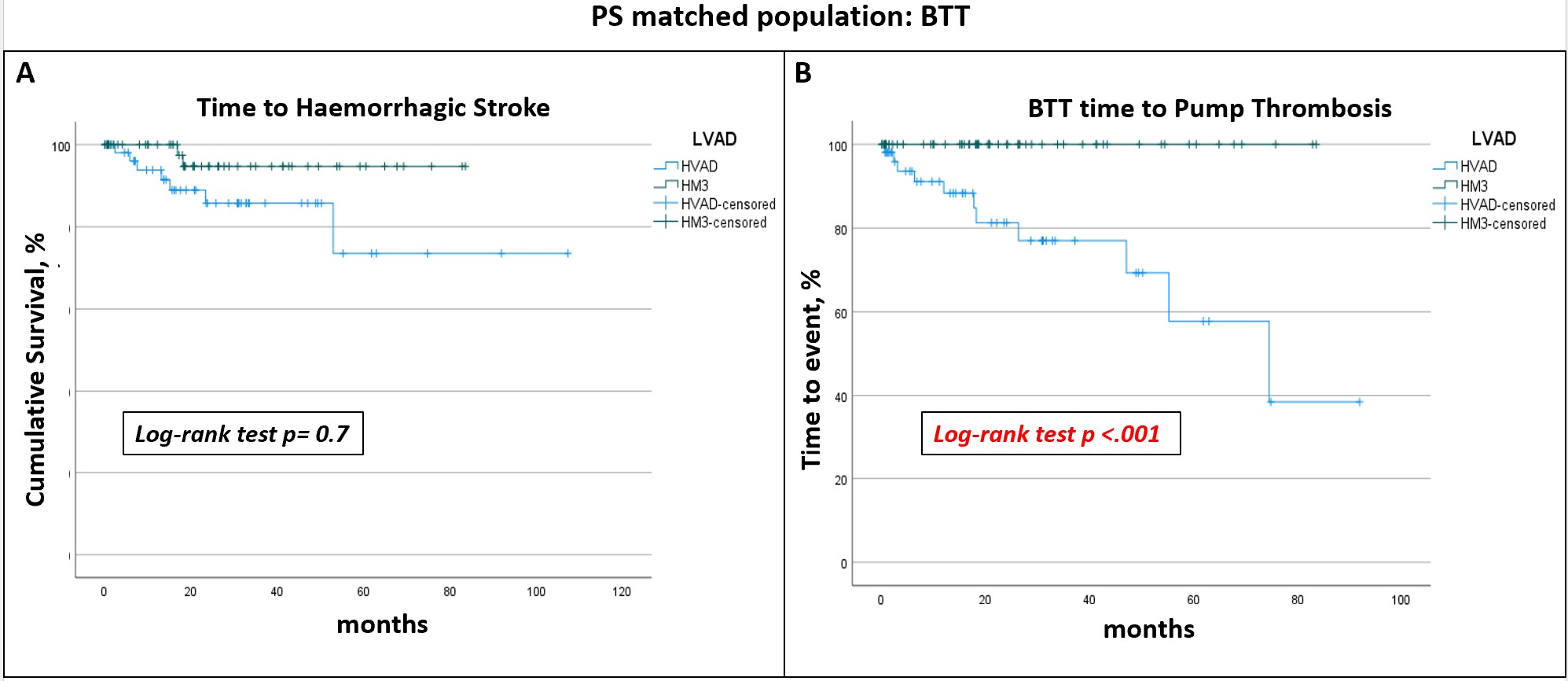

Supplement: Supplementary file 4 [file Image7.JPEG]

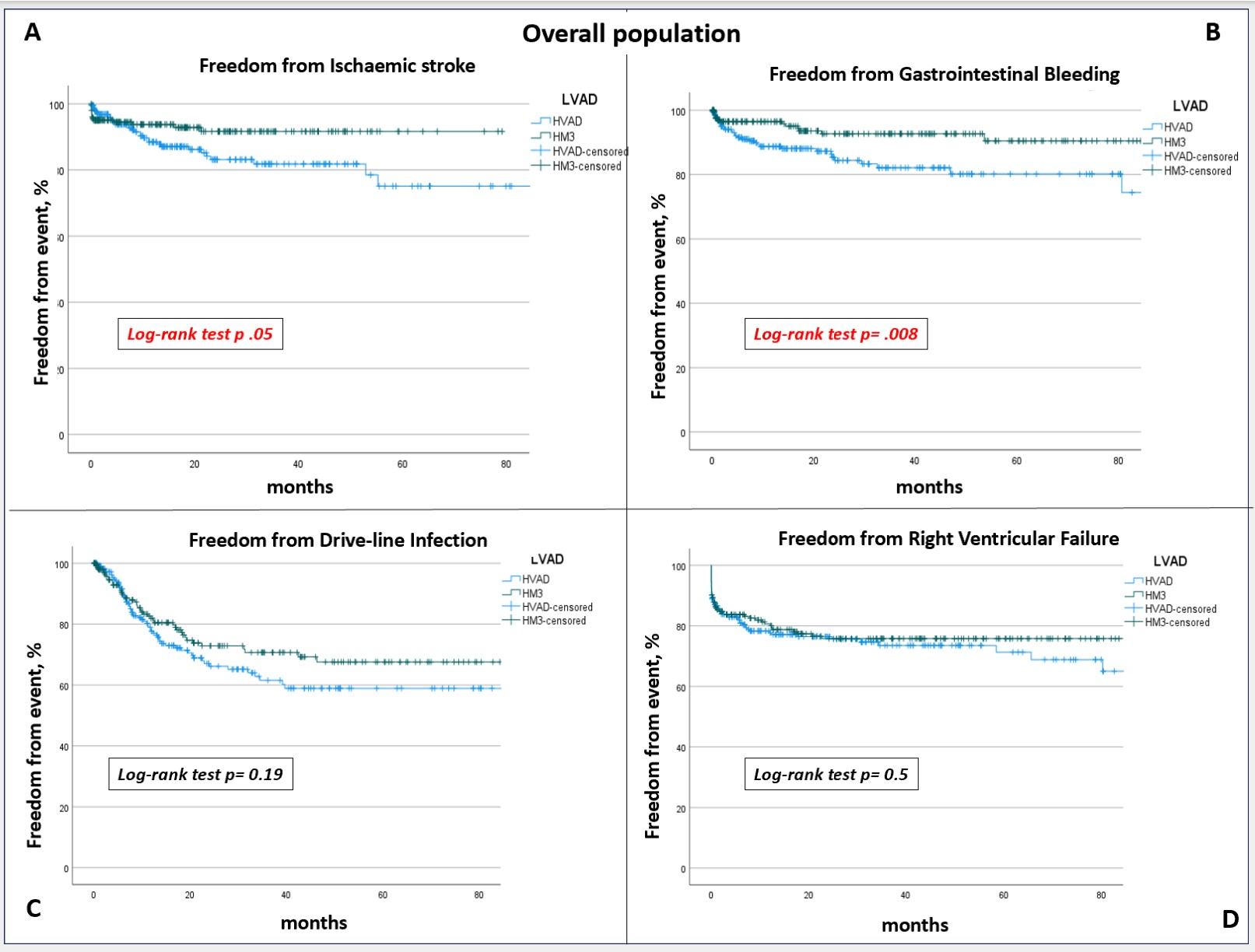

Supplement: Supplementary file 5 [file Image2.JPEG]

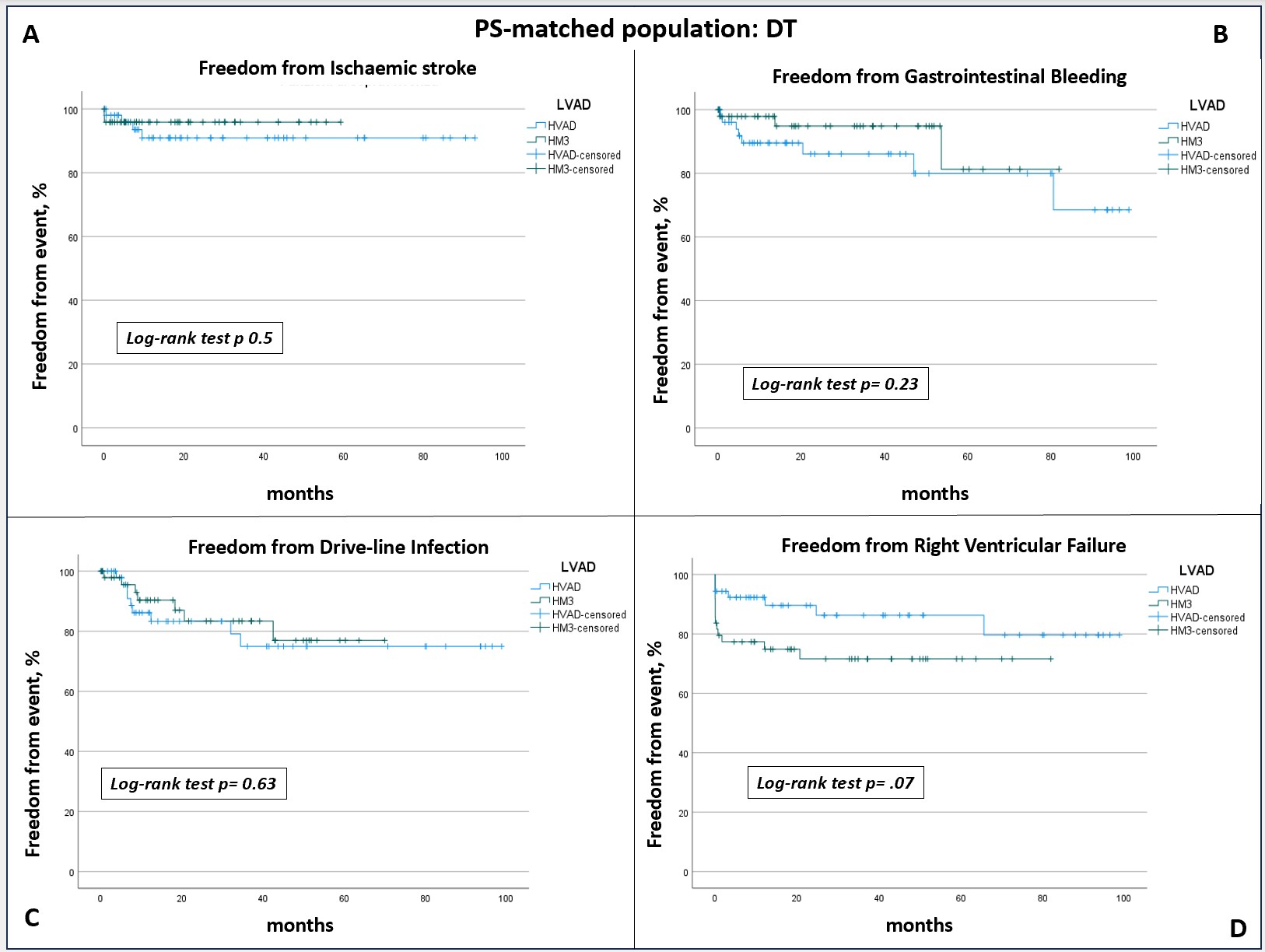

Supplement: Supplementary file 6 [file Image5.JPEG]

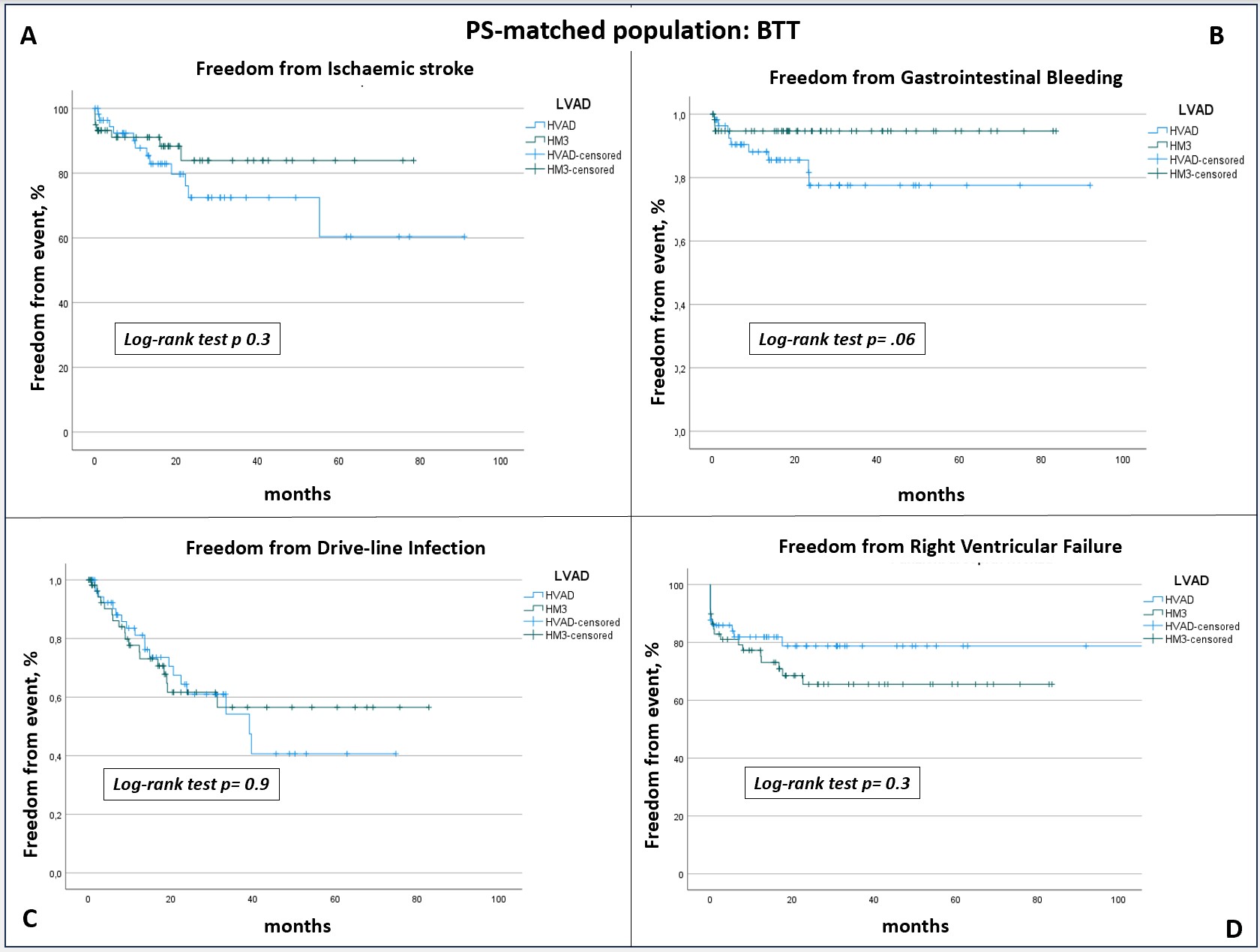

Supplement: Supplementary file 7 [file Image8.JPEG]

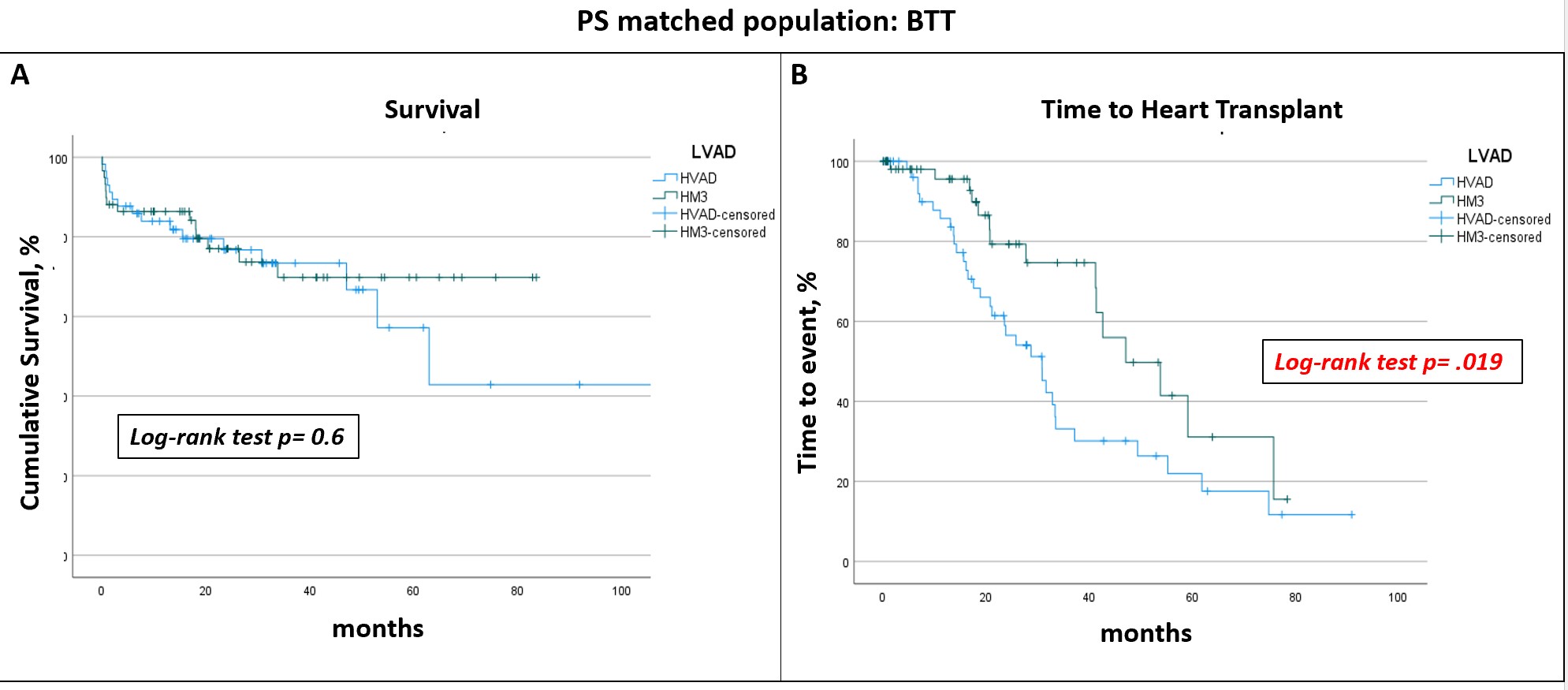

Supplement: Supplementary file 9 [file Image6.JPEG]
