# Supplementary material for: Five-Year Outcome After Continuous Flow LVAD With Full-Magnetic (HeartMate 3) Versus Hybrid Levitation System (HeartWare): A Propensity-Score Matched Study From an All-Comers Multicentre Registry
Source: Transpl Int. 2023 Sep 4;36:11675. doi: 10.3389/ti.2023.11675 (PMC10505657; doi:10.3389/ti.2023.11675)
Supplement: Supplementary file 8 [file DataSheet1.docx]

Supplemental Table 1. Overall population: Preoperative characteristics

| **Preoperative characteristics n (%), m (SD)** | **HM3**  **(n 214)** | **HVAD**  **(n 233)** | ***p*** |
| --- | --- | --- | --- |
| Age, years | 61.3 (9.08) | 58.07 (12.3) | *.002* |
| Sex, males | 192 (89.7) | 200 (85.8) | *0.21* |
| BSA, cm/m2 | 1.9 (0.19) | 1.8 (0.19) | *.003* |
| Creatinine, mg/dl | 1.5 (0.68) | 1.3 (0.51) | *.01* |
| AST,U/L | 42.7(82.2) | 52.8 (94.3) | *0.24* |
| ALT,U/L | 38.9 (60.7) | 70.4 (178.34) | *.017* |
| Atrial fibrillation | 52 (24.3) | 35 (15) | *.013* |
| EF,% | 20.9 (5.9) | 20.1 (6.7) | *0.2* |
| LVEDV,ml | 259.2 (77.6) | 262(102.62) 0,76 | *0.76* |
| TAPSE,mm | 16.9 (4.3) | 16.5 (4.4) | *0.47* |
| PVR (Fick),wood | 3.35 (2.05) | 3.6 (2.2) | *0.31* |
| Cardiac index (Fick) | 2.04 (0.61) | 1.9 (0.55) | *.004* |
| sPAP, mmHg | 48.4 (14.9) | 45.1 (16.7) | *.049* |
| Heart disease |  |  | *<.001* |
| Idiopathic | 79 (36.9) | 99 (42.5) |  |
| Hypertrophic | 26 (12.1) | 6 (2.6) |  |
| Ischemic | 101 (47.2) | 113 (48.5) |  |
| Other | 8 (1.8) | 15 (6.4) |  |
| INTERMACS |  |  | *0.18* |
| 1 | 21 (9.8) | 23 (9.8) |  |
| 2 | 43 (20.1) | 65 (27.9) |  |
| 3 | 107 (50) | 111 (47.6) |  |
| 4 | 43 (20.1) | 34 (14.6) |  |
| IABP | 79 (36.9) | 78 (52.1) | *0.4* |
| VA-ECMO | 19 (8.8) | 24 (10.3) | *0.6* |
| REDO | 11 (5.1) | 12 (5.2) | *0.9* |
| Indication |  |  | *0.34* |
| BTT | 74 (35.6) | 96 (41.2) |  |
| DT | 100 (46.7) | 96 (41.2) |  |
| BTC | 40 (18.7) | 41 (17.6) |  |

BSA, Body Surface Area; BTC, Bridge-to-Candidacy; BTT, Bridge-to-transplant; DT, Destination-Therapy; EF, Ejection Fraction; IABP, Intra-Aortic Balloon Pump; LVEDV; Left Ventricular End Diastolic Volume; PAP, systolic Pulmonary Arterial Pressure; PVR, Pulmonary Vascular Resistance; VA-ECMO, Veno-Arterial ExtraCorporeal Membrane Oxygenation

Supplemental Table 2. Overall population: In-hospital outcomes

| **Intra and post-operative variables**  **n (%), m (SD)** | **HM3**  **(n 214)** | **HVAD**  **(n 233)** | ***p*** |
| --- | --- | --- | --- |
| In-hospital outcome n (%), m (SD) | 30 (14) | 21 (9) | *0.28* |
| Median sternotomy | 209 (97.7) | 193 (82.8) | <.001 |
| Minimally invasive access | 5 (2.3) | 40 (17.2) | <.001 |
| CPB time, min | 107.5 (38.45) | 100 (43.7) | 0.14 |
| Off-pump implantation | 0 | 8 (3.4) | .008 |
| Total Implantation time,min | 326. 3(98.23) | 333.2 (217.4) | 0.7 |
| Bleeding requiring surgical revision | 21 (9.8) | 25 (10.7) | 0.75 |
| Prolonged ventilation (>72h) | 47 (22) | 23 (9.9) | <.001 |
| Dialysis | 36 (16.8) | 25 (10.7) | .061 |
| Sepsis | 74 (34.6) | 41(17.9) | <.001 |
| Ischaemic stroke | 10 (4.7) | 7 (3) | 0.36 |
| Haemorrhagic stroke | 1 (0.5) | 0 | 0.3 |
| Right ventricular failure | 31 (14.5) | 33 (14.2) | 0.92 |
| Temporary RVAD | 9 (4.2) | 6 (2.5) | 0.18 |
| ICU days | 19.3 (22.5) | 16.6 (22.14) | 0.25 |
| In-hospital days | 41.3 (42.8) | 38.6 (38.3) | 0.5 |

CPB, Cardiopulmonary Bypass; ICU, Intensive Care Unit; RVAD, Right Ventricular Assist Device

Supplemental Table 3. PS-matched population: BTT preoperative characteristics

| **Preoperative characteristics**  **n (%), m (SD)** | **HM3**  **(n 59)** | **HVAD**  **(n 53)** | ***p*** |
| --- | --- | --- | --- |
| Age, years | 56.1 (8.9) | 54.7 (8.6) | *0.43* |
| Sex, males | 53 (89.8) | 50 (94.3) | *0.38* |
| BSA, cm/m2 | 1.9 (0.19) | 1.9 (0.2) | *0.23* |
| Creatinine, mg/dl | 1.4 (0.49) | 1.4 (0.45) | *0.99* |
| AST,U/L | 36.3 (29.2) | 27.4 (21.6) | *0.27* |
| ALT,U/L | 41.2 (45.6) | 24.8 (14.9) | *0.14* |
| Atrial fibrillation | 14 (23.7) | 11(20.8) | *0.07* |
| EF,% | 21.3 (5.6) | 24.02 (7.4) | *.032* |
| LVEDV,ml | 265.4 (89.6) | 251.1 (112.4) | *0.55* |
| TAPSE,mm | 16.7 (4.9) | 16.7 (3.1) | *0.94* |
| PVR (Fick),wood | 3.4 (2.09) | 3.4 (2.07) | *0.97* |
| Cardiac index (Fick) | 1.89 (0.47) | 1.8 (0.46) | *0.93* |
| sPAP, mmHg | 49.07 (15.4) | 49.7 (15.7) | *0.83* |
| Heart disease |  |  | *0.33* |
| Idiopathic | 24 (45.3) | 31(43.6) |  |
| Hypertrophic | 1 (1.7) | 5(9,5)) |  |
| Ischemic | 24 (40.7) | 21 (39.6) |  |
| Other | 3 (5.4) | 3 (7.3) |  |
| INTERMACS |  |  | *0.91* |
| 1 | 5 (4.5) | 4 (10.4) |  |
| 2 | 10 (16.9) | 11 (27.1) |  |
| 3 | 30 (50.8) | 29 (54.7) |  |
| 4 | 14 (12.5) | 9 (8) |  |
| IABP | 17 (22.6) | 12 (28.8) | *0.45* |
| VA-ECMO | 3 (5.1) | 2 (3.8) | *0.73* |
| REDO | 2 (3.4) | 2 (387) | *0.9* |

BSA, Body Surface Area; BTT, Bridge-to-transplant; EF, Ejection Fraction; IABP, Intra-Aortic Balloon Pump; LVEDV; Left Ventricular End Diastolic Volume; PAP, systolic Pulmonary Arterial Pressure; PVR, Pulmonary Vascular Resistance; VA-ECMO, Veno-Arterial ExtraCorporeal Membrane Oxygenation

Supplemental Table 4. PS-matched population: BTT in-hospital outcomes

| **In-hospital outcome**  **n (%), m (SD)** | **HM3**  **(n 59)** | **HVAD**  **(n 53)** | ***p*** |
| --- | --- | --- | --- |
| In-hospital mortality | 8 (13.6) | 7 (13.2) | *0.96* |
| CPB time, min | 105.13 (41.6) | 90.8 (37.3) | *0.09* |
| Total Implantation time,min | 314.6 (80.9) | 240.3 (44.6) | *<001* |
| Bleeding requiring surgical revision | 9 (15.3) | 11 (20.8) | *0.45* |
| Prolonged ventilation (>72h) | 20 (33.9) | 6 (11.3) | *<.005* |
| Dialysis | 11 (18.6) | 6 (11.3) | *.28* |
| Sepsis | 23 (39) | 14 (26.4) | *.16* |
| Ischaemic stroke | 4 (6.8) | 3 (5.7) | *0.8* |
| Haemorrhagic stroke | 0 | 0 | *-* |
| Right ventricular failure | 11 (18.6) | 8 (15.1) | *0.62* |
| Temporary RVAD | 2 (15.4) | 2 (11.1) | *0.73* |
| ICU days | 16.5 (15.9) | 12.4 (14.2) | *0.15* |
| In-hospital days | 49.9 (69.19) | 44.5 (59.18) | *0.68* |

BTT, Bridge-to- Transplant; CPB, Cardiopulmonary Bypass; ICU, Intensive Care Unit; RVAD, Right Ventricular Assist Device
